# Dexamethasone and p38 MAPK inhibition of cytokine production from human lung fibroblasts

**DOI:** 10.1111/fcp.12627

**Published:** 2020-11-14

**Authors:** Andrew Higham, Dave Singh

**Affiliations:** ^1^ Division of Infection, Immunity and Respiratory Medicine School of Biological Sciences Faculty of Biology, Medicine and Health Manchester Academic Health Science Centre The University of Manchester and Manchester University NHS Foundation Trust Manchester UK; ^2^ Medicines Evaluation Unit Manchester UK

**Keywords:** COPD, inflammation, fibroblasts, inhaled corticosteroids, BIRB

## Abstract

Lung fibroblasts are involved in airway inflammation and remodelling in COPD. We report an investigation of the effects of combining a p38 MAPK inhibitor with a corticosteroid on cytokine production by a human lung fibroblast cell line and primary fibroblasts obtained from human lung tissue. Our main interest was to determine whether additive or synergistic anti‐inflammatory effects would be observed. We observed inhibition of IL‐6 and CXCL8 secretion from both lung fibroblast models by dexamethasone (maximal inhibition 40–90%) and the p38 MAPK inhibitor BIRB (maximal inhibition 30–60%), used alone and evidence of increased anti‐inflammatory effects when used in combination. This combination effect was more apparent for TNF‐a stimulated cytokine production (maximal inhibition increased by 10–20%). Interaction ratio analysis showed this enhanced effect to be additive rather than synergistic interaction. Similar results were obtained using both fibroblast cell culture models. Combining a p38 MAPK to corticosteroids may help reduce fibroblast mediated inflammation in COPD.

## Introduction

Fibroblasts are involved in airway remodelling in chronic obstructive pulmonary disease (COPD) [1]. These cells regulate the structure of the extracellular matrix (ECM) by secreting collagens, elastins and matrix metalloproteinases, which contribute to ECM turnover [2]. Fibroblasts also release pro‐inflammatory cytokines and chemokines which modify the activity of other inflammatory cells. Fibroblasts from COPD patients secrete higher levels of interleukin‐6 (IL‐6) and C‐X‐C motif chemokine ligand 8 (CXCL8) compared to healthy controls [3], which may contribute to the persistent inflammation in COPD lungs.

Inhaled corticosteroids (ICS) are commonly used anti‐inflammatory drugs in COPD. Corticosteroids suppress the binding of transcription factors such as nuclear factor‐kappa B (NF‐κB) to the promoter regions of inflammatory genes [4]. However, many COPD patients using ICS have persistent airway inflammation [5, 6] and continue to suffer with a heavy burden of symptoms and exacerbations [7].

P38 mitogen‐activated protein kinase (MAPK) activation increases the production of inflammatory mediators by enhancing gene transcription [8], mRNA stability and protein translation [9, 10]. Immunohistochemical analysis has shown increased p38 MAPK activation in the lungs of COPD patients [11, 12]. *In vitro* studies have demonstrated that p38 MAPK inhibitors reduce cytokine production from various cell types including alveolar macrophages, epithelial cells and lung lymphocytes [11, 13, 14, 15]. Furthermore, the combination of a p38 MAPK inhibitor with a corticosteroid synergistically enhances inhibition of cytokine production from alveolar macrophages and bronchial epithelial cells compared to corticosteroid alone [15, 16]. This may be related to phosphorylation of glucocorticoid receptor (GR) by p38 MAPK; p38 MAPK inhibition reduces phosphorylation of GR at serine 226, thereby increasing retention of GR in the nucleus and enhancing GR activity [15, 17]. Alternatively, corticosteroids increase the expression of phosphatases, including mitogen‐activated protein kinase phosphatase‐1 (MKP‐1) which downregulate p38 MAPK activity [18].

The effects of p38 MAPK inhibitors vary between different cell types [13]. This paper reports an investigation of the effects of combining a p38 MAPK inhibitor with a corticosteroid on cytokine production by pulmonary fibroblasts. Our main interest was to determine whether additive or synergistic anti‐inflammatory effects would be observed.

## Methods

### Subjects

Four patients undergoing surgical resection for suspected or confirmed lung cancer were recruited. There were two males and two females and all patients were smokers (3 current smokers and 1 ex‐smoker) without COPD (FEV_1_/FVC > 0.7). All subjects gave written informed consent. The study was approved by the local research ethics committee (South Manchester Research Ethics Committee).

### Primary human lung fibroblasts

Primary fibroblasts were isolated from resected lung tissue using the outgrowth technique [19]. Briefly, lung tissue was dissected into approximately 1mm^3^ pieces, rinsed twice in DMEM supplemented with Glutamax (Life Sciences), supplemented with 100 U/mL penicillin and 100 nm streptomycin (Sigma, Poole, UK) and 10% FCS (Life Sciences) and 3 pieces were placed into a 25 cm^2^ tissue culture flask and cultured in supplemented DMEM. Media was changed every 2–3 days until cells were 80% confluent. Primary fibroblasts were sub‐cultured using 0.05% trypsin‐EDTA (Invitrogen, Paisley, UK).

### Normal human lung fibroblasts

Normal human lung fibroblasts (NHLF) were purchased from Cambrex, UK. Fibroblasts were cultured in tissue culture flasks containing supplemented DMEM and media was changed every 2–3 days until cells were 80% confluent. Cells were sub‐cultured using 0.05% trypsin‐EDTA. All experiments using NHLFs were carried out in triplicate.

### Fibroblast cell culture

#### Cytokine analysis

Fibroblasts (primary and NHLFs) were seeded into flat bottomed 96 well plates at 25 × 10^5^ cells per well and grown until 80% confluent before serum starving overnight. Cells were then pre‐treated with dexamethasone (0.01–1 000 nm) (Sigma), BIRB‐796 (0.01–1 000 nm) (Stratech Scientific Ltd, Newmarket, UK), or vehicle control (dimethyl sulfoxide, Sigma) for one hour prior to stimulation with IL‐1β (10ng/ml, Peprotech, London, UK) or tumour necrosis factor‐α (TNF‐α) (50 ng/mL, Peprotech) for 24 h. In further experiments, each dexamethasone concentration was used in combination with each BIRB‐796 concentration. Supernatants for all experiments were harvested and stored at −80 °C. All experiments were carried out in triplicate. Cell culture supernatants were analysed by enzyme‐linked immunosorbent assays (ELISA) according to manufacturers’ instructions (R&D Systems, Abingdon Oxford) to quantify levels of CXCL8 and IL‐6. Lower limits of detection were 31.25 and 9.4 pg/mL respectively.

#### Western blot

NHLFs were seeded into flat bottomed 6 well plates at 1 × 10^6^ cells per well and grown until 80% confluent before serum starving overnight. Cells were then pre‐treated with dexamethasone (1 000 nm) or BIRB‐796 (1 000 nm) for one hour prior to stimulation with either IL‐1β (10 ng/mL) or TNF‐α (50 ng/mL) 0–60 min. Following stimulation, supernatants were removed and cells were rinsed with sterile PBS before being isolated in radio immunoprecipitation assay (RIPA) buffer (10 mm Tris‐HCl, pH 7.4, 150 mm NaCl, 1 mm EDTA, 0.1% Nonidet P‐40) containing phosphatase (Sigma Aldrich, Poole, Dorset, UK) and protease inhibitors (Calbiochem, San Diego, CA). The cell suspension was then centrifuged at 400 *g* for 10 min at 4 °C and protein quantification was performed on the supernatant using the Bradford Assay (Sigma). Samples were diluted to equal protein concentration in sample buffer (62.5 mm Tris, 10% glycerol, 1% SDS, 1% β‐mercaptoethanol and 0.01% bromphenol blue, pH 6.8), boiled at 90 °C and stored at −80 °C for western blot.

The following primary antibodies (from New England Biolabs, Hitchen, UK, unless stated) were used: p38 (#9212; recognizes p38α, ‐β, and ‐γ), pp38 (#9211; threonine 180 and tyrosine 182), GR (clone 41, Becton Dickinson UK, Oxford, UK) and pGR S211 (#4161).

Protein was electrophoresed on 10% SDS/acrylamide gels before being transferred onto 0.2 µm nitrocellulose membrane (BioRad, Hemel Hempstead, UK) for 1 h at 4 °C. Membranes were blocked for 1 h in blocking buffer (5% milk in 1× TBS, 0.1% tween‐20) before incubating with primary antibody (diluted in block buffer) over night at 4 °C. Membranes were washed for 2 × 5 min in wash buffer (88 mm Tris pH 7.8, 0.1% Tween‐20), prior to incubating with species‐specific horse radish peroxidise conjugated goat anti‐rabbit secondary antibody (New England Biolabs) for 1 h at room temperature. Membranes were washed (3 × 5 min) in wash buffer and immunoreactive proteins were visualized using enhanced chemiluminescence in the BioRad Universal Hood II with Quantity One Software. Protein molecular weights were determined using Precision Plus Standards (BioRad).

#### Immunofluorescence

Primary fibroblasts were seeded into flat bottomed 8 well glass chamber slides at 25 × 10^5^ cells per well and grown 2–3 days until 80% confluent. Media was removed and cells were washed in PBS before fixation in 4% paraformaldehyde. Cells were blocked in 1.5% heat inactivated normal serum (Vector Labs) then labelled with the following primary antibodies overnight at 4 °C: cytokeratin clone MNF116, alpha smooth muscle actin clone 1AF or vimentin clone Vim3B4 (all Dako). Primary antibodies were detected using Alexa 488 conjugated secondary antibodies (Invitrogen) and cells were counterstained with 4′, 6‐diamidino‐2‐phenylindole (DAPI).

### Statistics

Statistical analysis was performed using GraphPad Instat (GraphPad Software Inc, La Jolla, California, USA). Comparison of unstimulated to stimulated cells was analysed by a one‐way anova followed by a Dunnett’s multiple comparisons test. Dexamethasone and BIRB‐796 concentration curves were compared to their respective controls by a one‐way ANOVA followed by a Dunnett’s multiple comparison test. Comparison of dexamethasone and BIRB‐796 concentration curves between NHLFs and primary fibroblasts was analysed by a two‐way ANOVA followed by a Tukey’s multiple comparisons test. Comparison of combined dexamethasone and BIRB‐796 treatment to either compound alone was analysed by a two‐way ANOVA followed by a Tukey’s multiple comparison test. Interaction ratios were calculated from the ratio of observed efficacy (IO) to expected efficacy (IE). The expected efficacy was calculated using the Abbott formula: IE = A + B−(AB/100) where A is efficacy of compound A and B is efficacy of compound B. An interaction ratio between 0.5 and 1.5 is consistent with an additive effect [20].

## Results

### IL‐1β and TNF‐α stimulated CXCL8 and IL‐6 release from NHLFs and primary fibroblasts

#### NHLFs

**Figure 1:**
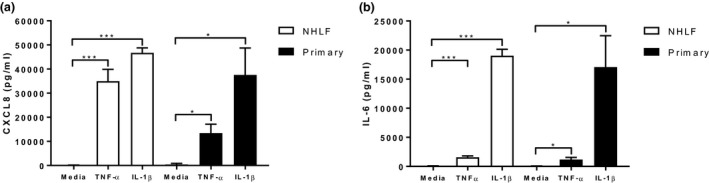
TNFα and IL‐1β induced CXCL8 and IL‐6 release from NHLFs and primary fibroblasts. NHLFs (*n* = 3) and primary human fibroblasts (*n* = 4) were cultured with and without IL‐1β and TNFα for 24 h and supernatants were collected and analysed for CXCL8 (a) and IL‐6 (b) release. Data shown are mean ± SEM. * and *** = significant difference compared to untreated control where *P* < 0.05 and *P* < 0.001 respectively.

In NHLFs, TNF‐α significantly increased release of CXCL8 and IL‐6 (both *P* < 0.001) compared to basal levels (*Figure* *1*). Similarly, IL‐1β significantly increased CXCL8 and IL‐6 (both *P* < 0.001) release compared to basal levels (*Figure* *1*). IL‐1β stimulated significantly higher levels of IL‐6 release compared to TNF‐α (*P* < 0.001).

#### Primary fibroblasts

Primary fibroblast cultures were not contaminated with epithelial cells; the cells were positive for the fibroblast markers vimentin and α‐smooth muscle actin and negative for the epithelial marker cytokeratin (*Figure* S1).

In primary fibroblasts, TNF‐α significantly increased secretion of CXCL8 and IL‐6 compared to basal levels (*P* = 0.03 and *P* = 0.04 respectively; *Figure* *1*). Similarly, IL‐1β significantly increased CXCL8 and IL‐6 release compared to basal levels (*P* = 0.04 and *P* = 0.03 respectively). There was a trend towards significantly higher IL‐1β stimulated release of IL‐6 compared to TNF‐α (*P* = 0.06).

TNF‐α stimulated CXCL8 release from NHLFs was significantly higher compared to primary fibroblasts (*P* = 0.02; *Figure* *1*). There was no significant difference in the levels of TNF‐α stimulated IL‐6 and IL‐1β stimulated CXCL8 and IL‐6 when comparing NHLFs to primary fibroblasts.

### TNF‐α and IL‐1β stimulate phosphorylation of p38 MAPK in NHLFs

In NHLFs, TNF‐α and IL‐1β caused a time dependent increase in the phosphorylation of p38‐MAPK, returning to basal levels by 60 min (*Figure* S2). Neither TNF‐α or IL‐1β changed total p38‐MAPK expression.

**Figure 2:**
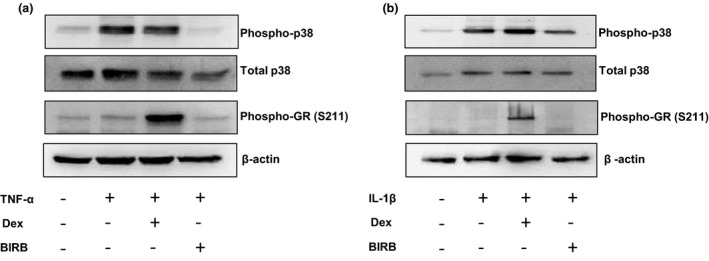
The effects of dexamethasone and BIRB‐796 on phospho‐p38 MAPK expression in NHLFs. NHLFs (*n* = 3) were pre‐treated with dexamethasone (1 000 nm) or BIRB‐796 (1 000 nm) for 1 h prior to stimulation with TNF‐α (a) or IL‐1β (b) for 20 min. Cell lysates were analysed for phospho‐p38 MAPK, total p38,phosphor‐GR (S211)and beta actin by western blot.

Dexamethasone (1 000 nm) caused a time dependant increase in glucocorticoid receptor (GR) phosphorylation at serine 211 in NHLFs (*Figure* S2). Dexamethasone (1 000 nm) did not inhibit IL‐1β or TNF‐α stimulated phosphorylation of p38 MAPK (*Figure* *2*). In contrast, BIRB‐796 (1000nM) inhibited IL‐1β and TNF‐α stimulated phosphorylation of p38 MAPK.

### Dexamethasone inhibition of cytokine release

**Figure 3:**
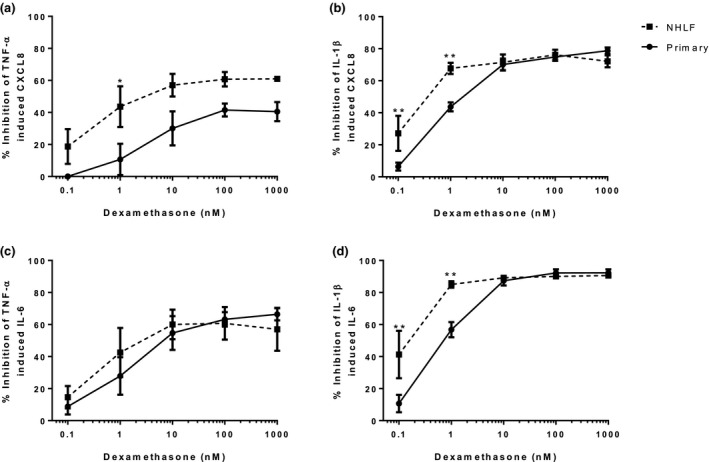
Dexamethasone inhibition of TNF‐α and IL‐1β induced CXCL8 and IL‐6 release from NHLFs and primary fibroblasts; per cent inhibition. NHLFs (*n* = 3) and primary (*n* = 4) fibroblasts were pre‐treated with dexamethasone (0.1–1000 nm) for 1 h prior to TNF‐α (a and c) or IL‐1β (b and d) stimulation. Supernatants were analysed for CXCL8 (A and B) and IL‐6 (c and d). Data shown are mean ± SEM. * and ** = significant difference between NHLFs and primary fibroblasts where*P* < 0.05 and *P* < 0.01 respectively

In NHLFs and primary fibroblasts, dexamethasone significantly inhibited TNF‐α and IL‐1β stimulated CXCL8 and IL‐6 release in a concentration dependant manner (*Figure* *3* and *Figure* S3). Dexamethasone inhibition of TNF‐α stimulated CXCL8 (at 1 nm) and IL‐1β stimulated CXCL8 and IL‐6 (at 0.1 and 1 nm) release was significantly greater (*P* < 0.05) in NHLFs compared to primary fibroblasts.

### P38 MAPK inhibition of cytokine release

**Figure 4:**
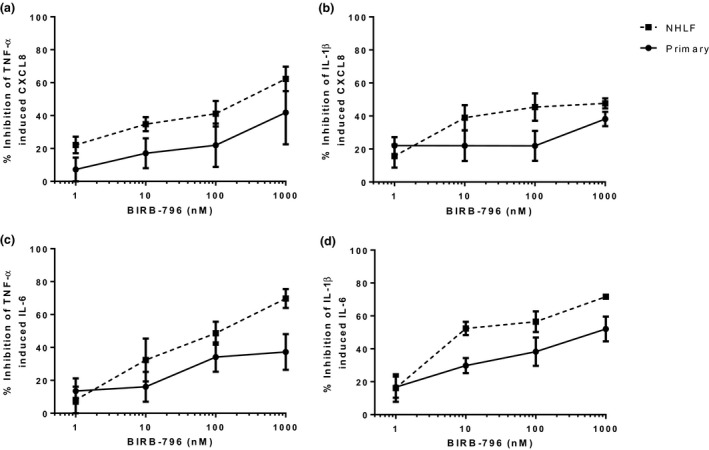
BIRB‐796 inhibition of TNF‐α and IL‐1β induced CXCL8 and IL‐6 release from NHLFs and primary fibroblasts; per cent inhibition. NHLFs (*n* = 3) and primary fibroblasts (*n* = 4) were pre‐treated with BIRB‐796 (0.1–1000 nm) for 1 h prior to TNF‐α (a and c) or IL‐1β (b and d) stimulation. Supernatants were analysed for CXCL8 (a and b) and IL‐6 (c and d). Data shown are mean ± SEM.

In NHLFs and primary fibroblasts, BIRB‐796 inhibited TNF‐α and IL‐1β stimulated CXCL8 and IL‐6 release in a concentration dependant manner (*Figures* *4* and S4). However, in primary fibroblasts, the level of inhibition did not reach statistical significance for TNF‐α stimulated CXCL8 release. There was no significant difference in the level of BIRB‐796 inhibition when comparing NHLFs to primary fibroblasts.

### Combination Inhibition of cytokine release

**Figure 5:**
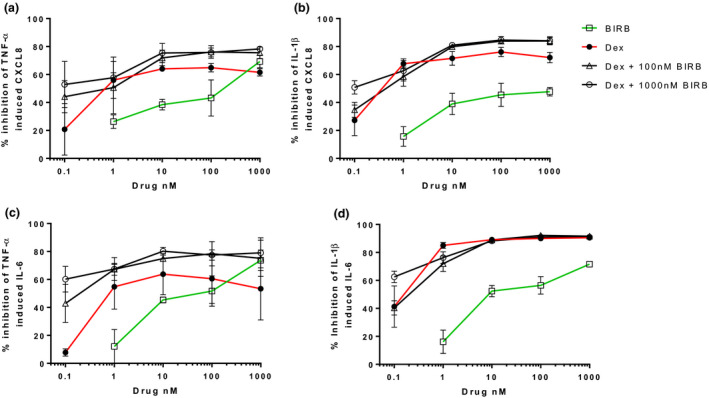
Combined dexamethasone and BIRB‐796 inhibition of TNF‐α and IL‐1β induced CXCL8 and IL‐6 from NHLFs. NHLFs (*n* = 3) were pre‐treated with dexamethasone (0.1–1 000 nm), or BIRB‐796 (0.1–1 000 nm), in combination for 1 h prior to TNF‐α (a and c) or IL‐1β (b and d) stimulation. Supernatants were analysed for CXCL8 (a and b) and IL‐6 (c and d). Data shown are mean ± SEM.

**Figure 6:**
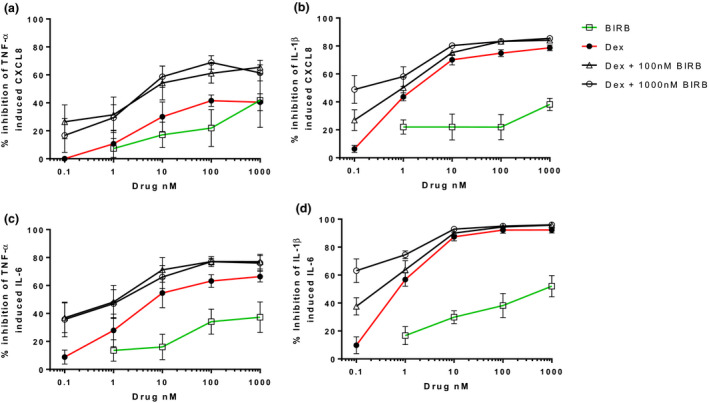
Combined dexamethasone and BIRB‐796 inhibition of TNF‐α and IL‐1β induced CXCL8 and IL‐6 release from primary fibroblasts. Primary fibroblasts (*n* = 4) were pre‐treated with dexamethasone (0.1–1 000 nm), or BIRB‐796 (0.1–1 000 nm), in combination for 1 hour prior to TNF‐α (a and c) or IL‐1β (b and d) stimulation. Supernatants were analysed for CXCL8 (a and b) and IL‐6 (c and d). Data shown are mean ± SEM.

In NHLFs and primary fibroblasts, the effects of combination treatment were numerically greater than either treatment alone (*Figures* *5*, *6* and *5*, *6*), although these differences were more apparent for TNF‐α stimulated cytokines than IL‐1β stimulated cytokines. Statistically significant differences for combination treatment versus one treatment were observed at a number of concentrations (see *Table* *1* for the highest drug concentrations and *Tables* S1 *–* S4 for complete listing of results). For the majority of combinations, interaction ratios ranged from 0.5 to 1.2 with none over 1.5, indicating that increased inhibition by combining dexamethasone with BIRB‐796 was addition and not synergy.

**Table 1:** Per cent inhibition of TNF‐α and IL‐1β induced cytokine production from NHLFs and primary fibroblasts by dexamethasone and BIRB‐796 alone or in combination

	TNF‐α induced CXCL8		IL‐1β induced CXCL8		TNF‐α induced IL‐6		IL‐1β induced IL‐6	
	NHLF	Primary	NHLF	Primary	NHLF	Primary	NHLF	Primary
Dex (1 000 nm)	61%	40%	72%***	79%***	57%	66%***	91%**	92%***
BIRB (1 000 nm)	62%	42%	47%	38%	70%	37%	72%	52%
Dex (1 000 nm) + BIRB (100 nm)	78%†	61%	84%† ^¶¶¶^	85%^¶¶¶^	79%	76%^¶¶¶^	91% ^¶¶¶^	96%^¶¶¶^
IR	0.9	0.9	1.0	1.0	0.9	1.0	0.9	1.0

Data presented as means. *, **, *** = significantly above BIRB‐796 (2‐way anova where *P* < 0.05, 0.01 and 0.001 respectively). † = significantly above dexamethasone (2‐way anova where *P* < 0.05). ¶¶¶ = significantly above BIRB‐796 (2‐way anova where *P* < 0.001). Dex: dexamethasone; IR: interaction ratio.

## Discussion

We have demonstrated inhibition of lung fibroblast cytokine secretion by a corticosteroid or a p38 MAPK inhibitor used alone, and increased anti‐inflammatory effects when used in combination. This combination effect was more apparent for TNF‐α stimulated cytokine production. Interaction ratio analysis showed this enhanced effect to be additive rather than synergistic interaction.

A strength of this study is the use of two different sources of lung fibroblasts; a cell line and fibroblasts cultured directly from surgically resected lungs. We observed similar results with both cell types concerning additive combination effects. It has previously been shown that increased p38 MAPK phosphorylation is associated with reduced sensitivity to corticosteroids [14, 15, 21]. We and others have shown that p38 MAPK inhibition reduces phosphorylation of GR at serine 226 [15, 22]; phosphorylation at serine 226 causes GR to shuttle out of the nucleus, thereby reducing GR activity [21, 23]. By inhibiting p38 MAPK, GR remains in the nucleus for longer enabling prolonged suppression of gene transcription [24]. This is a molecular mechanism to explain the synergistic effects of combined corticosteroid and p38 MAPK inhibitor treatment [15, 16]. We did not observe a synergistic effect of combined treatment. Nevertheless, our results suggest that useful additional anti‐inflammatory effects on lung fibroblasts can be obtained by combining these two drug classes that act through different mechanisms.

We observed greater additive effects in the TNF‐α stimulated compared to the IL‐1β stimulated cellular models. TNF‐α and IL‐1β production and signal transduction are different processes. Following exposure to pathogen‐associated molecular patterns (PAMPs) or danger‐associated molecular patterns (DAMPs), de novo synthesis of TNF‐α occurs and membrane bound TNF‐α is cleaved by TNF‐α converting enzyme (TACE) to produce soluble TNF‐α which activates TNF receptor 1 (TNFR1) or TNFR2 [25]. Conversely, IL‐1β secretion is a two part process: firstly, pro‐IL‐1β is produced in response to an initial PAMP/DAMP signal, considered a priming event. Upon exposure to a second signal, pro‐IL‐1β is processed to its mature form by caspase‐1 at the inflammasome and secreted to activate the IL‐1 receptor/ IL‐1 receptor accessory protein complex [26]. Both TNF‐α and IL‐1β signalling converge on similar downstream effectors including NF‐κB, activator protein‐1 (AP‐1) and p38 MAPK, to induce pro‐inflammatory gene transcription [25, 26]. However, differences in the downstream signalling cascade have been observed, specifically TNF‐α dependant activation of interferon regulatory factor (IRF) 3 and IRF7 [27]. This may reflect temporal differences in post‐transcriptional programs and sustained anti‐viral responses which contribute to higher levels of inflammatory cytokine output.

Our results indicate that IL‐1β signalling, compared to TNF‐α signalling, is more sensitive to corticosteroid inhibition in human lung fibroblasts. This may be related to differences in the signalling pathways outlined above. TNF‐α is a pro‐inflammatory cytokine which amplifies the immune response and increases leucocyte influx [28]. The levels of TNF‐α are increased in the airways of COPD patients compared to controls and in the sputum of exacerbating COPD patients compared to the stable state [29, 30]. Bacterial endotoxin is a potent stimuli of TNF‐α from various cell types including lung macrophages [31]. Increased levels of TNF‐α are increased in the airways of exacerbating COPD patients colonized with *Non typeable Haemophilus influenza* and *Moraxella catarrhalis* [32]. Bacterial colonization of the airways is known to cause inflammation and tissue remodelling in COPD patients [1, 33, 34], and TNF‐α appears to have a central role in these processes. Our results suggest that combining a p38 MAPK inhibitor with a corticosteroid can be an effective anti‐inflammatory strategy that targets TNF‐α driven signalling pathways in human lung fibroblasts.

CXCL8 is a neutrophil chemokine that is less sensitive to corticosteroid mediated inhibition compared to other cytokines released from lung macrophages and smooth muscle cells [35, 36]. CXCL8 levels are increased in the lungs of COPD patients [37, 38]. Uncontrolled influx of neutrophils during chronic inflammation can cause tissue damage due to the release of proteases [39, 40]. The addition of a p38 MAPK inhibitor to corticosteroid treatment may therefore enhance inhibition of CXCL8 production from various cell types including fibroblasts, therefore limiting the damage caused by neutrophil mediated inflammation [15, 16, 17].

It has previously been shown that the p38 MAPK inhibitor SB203580 reduces bradykinin induced production of IL‐6 and CXCL8 from primary human lung fibroblasts and IL‐1β and TNF‐α induced IL‐6 and CXCL8 from synovial fibroblasts [41, 42]. However, significant inhibition was only observed at high concentrations of compound (1–30 µm). We observed inhibition at concentrations as low as 0.1 nm, using a different p38 MAPK inhibitor. Although not studied here, p38 MAPK inhibition reduces fibroblast proliferation and differentiation into myofibroblasts [43]. An increase in fibroblast numbers and differentiation into myofibroblasts can lead to scarring and fibrotic remodelling [1]. These findings may have implications for fibrotic remodelling of the small airways, a common feature in small airway disease in COPD [44].

We confirmed the identity of primary fibroblasts by positive immunoreactivity for the fibroblast marker, vimentin [45]. We also observed immunoreactivity for α‐smooth muscle actin, used to identify activated fibroblasts or differentiation to myofibroblasts [45]. Samples were obtained from donors with a smoking history and thus, previous smoke exposure may have influenced the characteristics of the isolated fibroblasts; cigarette smoke increases myofibroblast differentiation [46, 47]. However, we cannot rule out the impact of culture conditions (e.g. time, cultureware) on the expression of α‐smooth muscle actin; cardiac fibroblasts express lower levels of α‐smooth muscle actin when cultured on collagen coated pads compared to standard cultureware [48]. Nevertheless, the use of activated fibroblasts in our culture system is highly relevant as this may better represent the status of these cells in vivo.

Clinical trials using p38 MAPK inhibitors in COPD patients have yielded mixed results [49, 50, 51]. However, narrow spectrum p38 MAPK inhibitors and targeted suppression of inflammation post exacerbation have shown promise [52, 53, 54]. COPD exacerbations contribute to inflammation and remodelling of small airways in COPD patients. P38 MAPK inhibition may therefore reduce pro‐inflammatory and pro‐fibrotic effects of lung fibroblasts which contribute to small airway disease.

## Conclusion

In conclusion, we have demonstrated additional anti‐inflammatory effects when combining a p38 MAPK inhibitor with a corticosteroid in human lung fibroblasts. This may have implications for fibroblast mediated inflammation and remodelling in COPD.

## Conflicts of interest

AH has received personal fees from Chiesi. DS has received personal fees from AstraZeneca, Boehringer Ingelheim, Chiesi, Cipla, GlaxoSmithKline, Glenmark, Menarini, Mundipharma, Novartis, Peptinnovate, Pfizer, Pulmatrix, Therevance and Verona.

## Funding

This research was supported by the NIHR Manchester Biomedical Research Centre and the North West Lung Centre Charity, Manchester. This report is independent research and the views expressed in this publication are those of the authors and not necessarily those of the NHS, the NIHR or the Department of Health.

## Supporting information

Figure S1 Primary fibroblast identification.

Figure S2 Dexamethasone inhibition of TNFα and IL‐1β induced CXCL8 and IL‐6 release from NHLFs and primary fibroblasts; absolute values.

Figure S3 BIRB‐796 inhibition of TNFα and IL‐1β induced CXCL8 and IL‐6 release from NHLFs and primary fibroblasts; absolute values.

Figure S4 Time course of phospho‐38 MAPK and phospho‐GR (S211) expression in NHLFs.

Table S1 Percent inhibition of TNF‐α induced CXCL8 production from NHLFs and primary fibroblasts by dexamethasone and BIRB‐796 alone or in combination.
Table S2 Percent inhibition of TNF‐α induced IL‐6 production from NHLFs and primary fibroblasts by dexamethasone and BIRB‐796 alone or in combination
Table S3 Percent inhibition of IL‐1β induced CXCL8 production from NHLFs and primary fibroblasts by dexamethasone and BIRB‐796 alone or in combination.
Table S4 Percent inhibition of IL‐1β induced CXCL8 production from NHLFs and primary fibroblasts by dexamethasone and BIRB‐796 alone or in combination.
