# Supplementary figures and images for: Dexamethasone and p38 MAPK inhibition of cytokine production from human lung fibroblasts
Source: Fundam Clin Pharmacol. 2020 Nov 14;35(4):714–24. doi: 10.1111/fcp.12627 (PMC8451891; doi:10.1111/fcp.12627)

Supplementary Figure 1

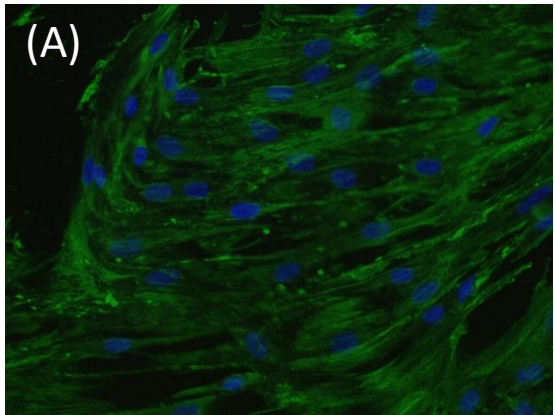

Vimentin

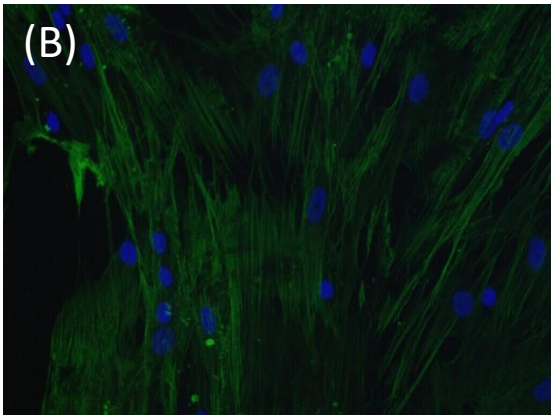

$\alpha$ -smooth muscle actin

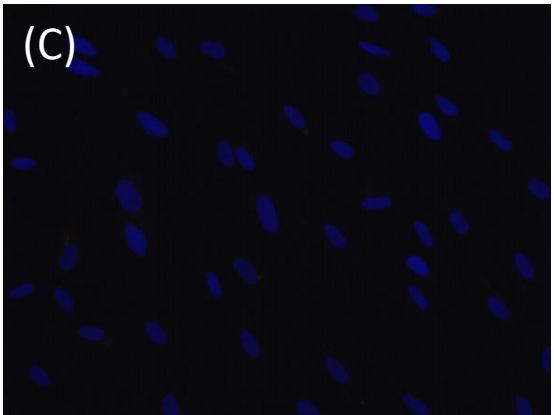

Cytokeratin

Supplement: Supplementary file 1 — Figure S1 Primary fibroblast identification. [file FCP-35-714-s002.pdf]

Supplementary Figure 2

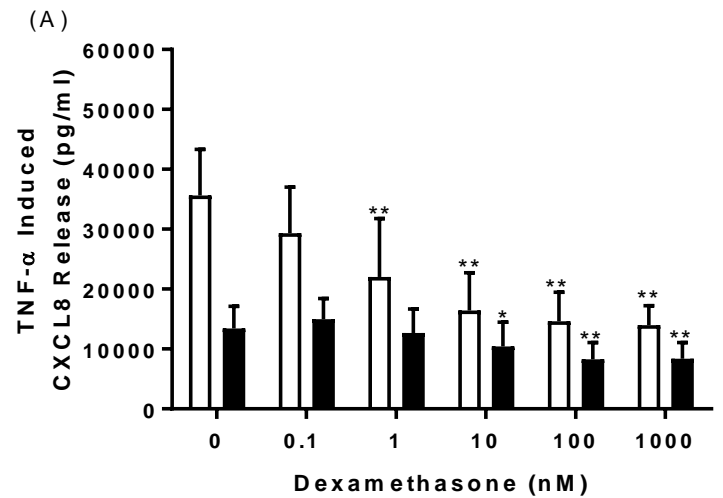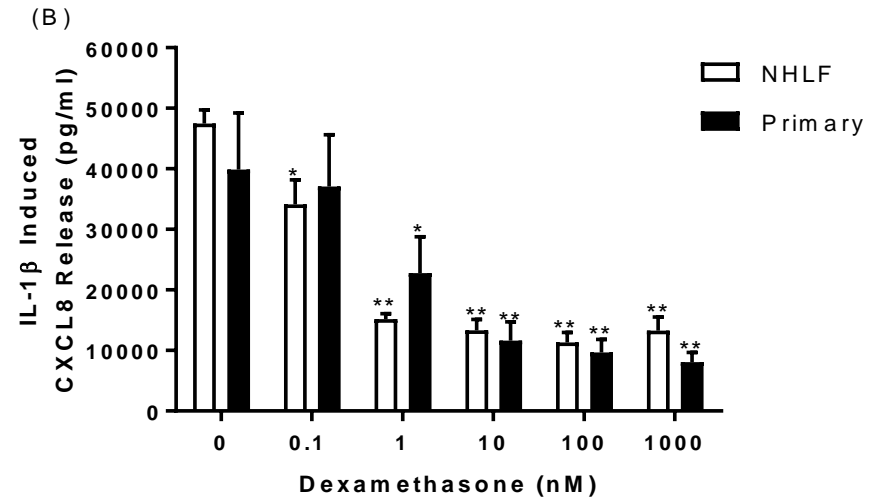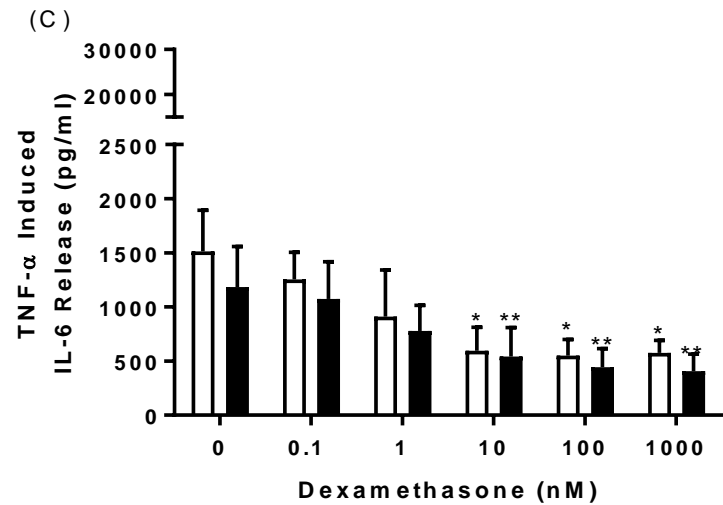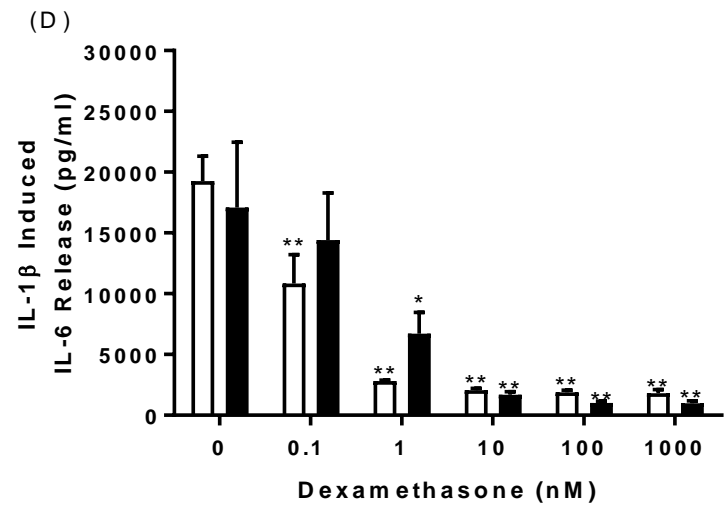

Supplement: Supplementary file 2 — Figure S2 Dexamethasone inhibition of TNFα and IL‐1β induced CXCL8 and IL‐6 release from NHLFs and primary fibroblasts; absolute values. [file FCP-35-714-s005.pdf]

Supplementary Figure 3

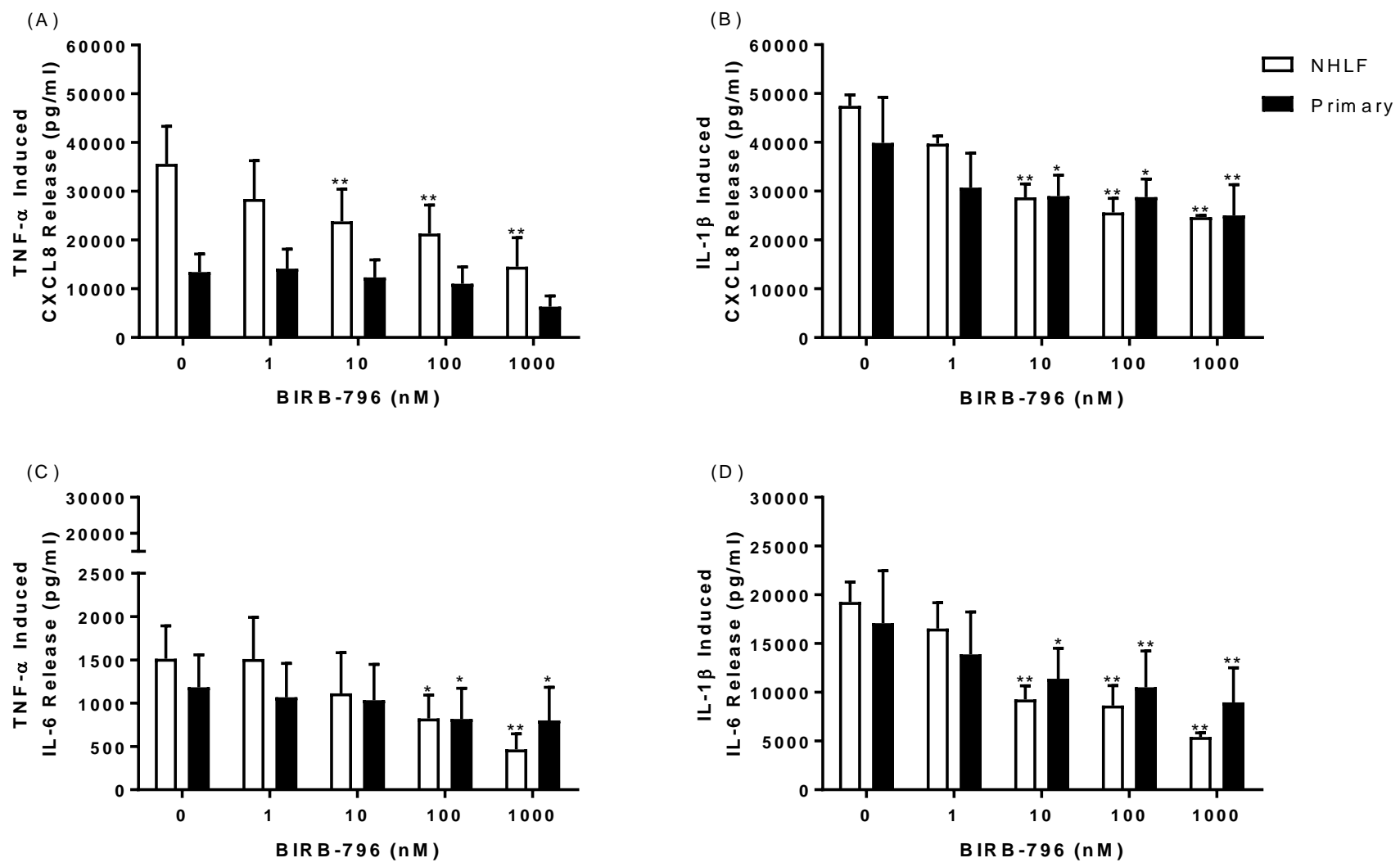

Supplement: Supplementary file 3 — Figure S3 BIRB‐796 inhibition of TNFα and IL‐1β induced CXCL8 and IL‐6 release from NHLFs and primary fibroblasts; absolute values. [file FCP-35-714-s001.pdf]

Supplementary Figure 4

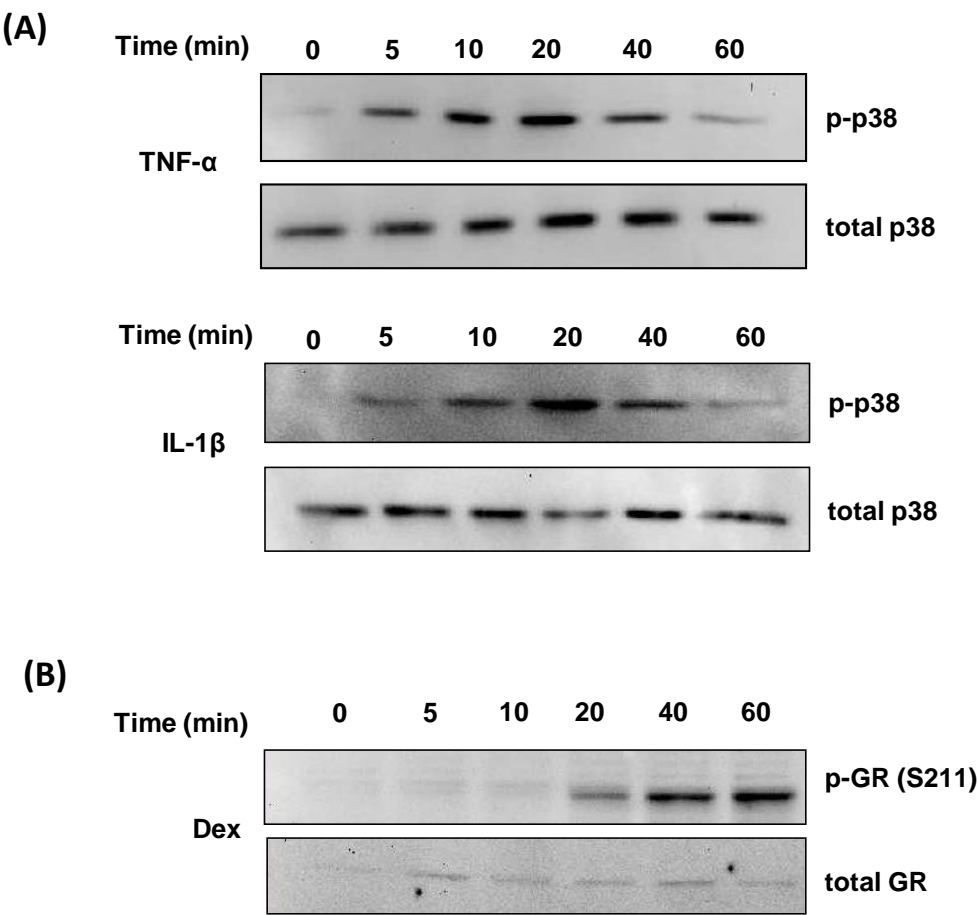

Supplement: Supplementary file 4 — Figure S4 Time course of phospho‐38 MAPK and phospho‐GR (S211) expression in NHLFs. [file FCP-35-714-s004.pdf]
