# Supplementary material for: Dexamethasone and p38 MAPK inhibition of cytokine production from human lung fibroblasts
Source: Fundam Clin Pharmacol. 2020 Nov 14;35(4):714–24. doi: 10.1111/fcp.12627 (PMC8451891; doi:10.1111/fcp.12627)
Supplement: Supplementary file 5 — Table S1 Percent inhibition of TNF‐α induced CXCL8 production from NHLFs and primary fibroblasts by dexamethasone and BIRB‐796 alone or in combination. Table S2 Percent inhibition of TNF‐α induced IL‐6 production from NHLFs and primary fibroblasts by dexamethasone and BIRB‐796 alone or in combination Table S3 Percent inhibition of IL‐1β induced CXCL8 production from NHLFs and primary fibroblasts by dexamethasone and BIRB‐796 alone or in combination. Table S4 Percent inhibition of IL‐1β induced CXCL8 production from NHLFs and primary fibroblasts by dexamethasone and BIRB‐796 alone or in combination. [file FCP-35-714-s003.docx]

| **Condition** | **NHLF** | | **Primary** | |
| --- | --- | --- | --- | --- |
|  | **% Inhibition** | **Interaction Ratio** | **% Inhibition** | **Interaction Ratio** |
| Dex 0.1nM | 21 | N/A | 0 | N/A |
| Dex 0.1 nM + BIRB 100 nM | 44 | 0.8 | 26 | 1.2 |
| Dex 0.1 nM + BIRB 1000 nM | 53 | 0.7 | 17 | 0.4 |
| Dex 1nM | 56 | N/A | 11 | N/A |
| Dex 1 nM + BIRB 100 nM | 51 | 0.7 | 31 | 1.0 |
| Dex 1 nM + BIRB 1000 nM | 58 | 0.7 | 29 | 0.6 |
| Dex 10 nM | 64 | N/A | 30 | N/A |
| Dex 10 nM + BIRB 100 nM | 72 | 0.9 | 54 | 1.2 |
| Dex 10 nM + BIRB 1000 nM | 75 | 0.8 | 59 | 1.0 |
| Dex 100 nM | 65 | N/A | 42 | N/A |
| Dex 100 nM + BIRB 100 nM | 76 | 0.9 | 61 | 1.1 |
| Dex 100 nM + BIRB 1000 nM | 76 | 0.9 | 69 | 1.0 |
| Dex 1000 nM | 62 | N/A | 40 | N/A |
| Dex 1000 nM + BIRB 100 nM | 76 | 1.0 | 65 | 1.2 |
| Dex 1000 nM + BIRB 1000 nM | 78 | 0.9 | 61 | 0.9 |

**Supplementary table 1.** Percent inhibition of TNF-α induced CXCL8 production from NHLFs and primary fibroblasts by dexamethasone and BIRB-796 alone or in combination.

Data presented as means.

**Supplementary table 2.** Percent inhibition of TNF-α induced IL-6 production from NHLFs and primary fibroblasts by dexamethasone and BIRB-796 alone or in combination.

| **Condition** | **NHLF** | | **Primary** | |
| --- | --- | --- | --- | --- |
|  | **% Inhibition** | **Interaction Ratio** | **% Inhibition** | **Interaction Ratio** |
| Dex 0.1nM | 8 | N/A | 9 | N/A |
| Dex 0.1 nM + BIRB 100 nM | 43 | 0.8 | 36 | 0.9 |
| Dex 0.1 nM + BIRB 1000 nM | 60* | 0.8 | 36 | 0.8 |
| Dex 1nM | 55 | N/A | 28 | N/A |
| Dex 1 nM + BIRB 100 nM | 67 | 0.9 | 48* | 0.9 |
| Dex 1 nM + BIRB 1000 nM | 67 | 0.8 | 47* | 0.9 |
| Dex 10 nM | 64 | N/A | 55 | N/A |
| Dex 10 nM + BIRB 100 nM | 75 | 0.9 | 71 | 1.0 |
| Dex 10 nM + BIRB 1000 nM | 80 | 0.9 | 66 | 0.9 |
| Dex 100 nM | 61 | N/A | 63 | N/A |
| Dex 100 nM + BIRB 100 nM | 78 | 1.0 | 77 | 1.0 |
| Dex 100 nM + BIRB 1000 nM | 77 | 0.9 | 77 | 1.0 |
| Dex 1000 nM | 53 | N/A | 66 | N/A |
| Dex 1000 nM + BIRB 100 nM | 75* | 1.0 | 77 | 1.0 |
| Dex 1000 nM + BIRB 1000 nM | 79** | 0.9 | 76 | 1.0 |

Data presented as means. *, ** = significantly above dex alone (2-way ANOVA where p<0.05, and 0.01 respectively).

**Supplementary table 3.** Percent inhibition of IL-1β induced CXCL8 production from NHLFs and primary fibroblasts by dexamethasone and BIRB-796 alone or in combination.

| **Condition** | **NHLF** | | **Primary** | |
| --- | --- | --- | --- | --- |
|  | **% Inhibition** | **Interaction Ratio** | **% Inhibition** | **Interaction Ratio** |
| Dex 0.1nM | 27 | N/A | 6 | N/A |
| Dex 0.1 nM + BIRB 100 nM | 35 | 0.6 | 27* | 1.0 |
| Dex 0.1 nM + BIRB 1000 nM | 51** | 0.8 | 49*** | 1.2 |
| Dex 1nM | 68 | N/A | 44 | N/A |
| Dex 1 nM + BIRB 100 nM | 58 | 0.7 | 50 | 0.9 |
| Dex 1 nM + BIRB 1000 nM | 63 | 0.8 | 58 | 0.9 |
| Dex 10 nM | 72 | N/A | 70 | N/A |
| Dex 10 nM + BIRB 100 nM | 80 | 0.9 | 75 | 1.0 |
| Dex 10 nM + BIRB 1000 nM | 81 | 0.9 | 80 | 1.0 |
| Dex 100 nM | 76 | N/A | 75 | N/A |
| Dex 100 nM + BIRB 100 nM | 84 | 1.0 | 83 | 1.0 |
| Dex 100 nM + BIRB 1000 nM | 85 | 1.0 | 83 | 1.0 |
| Dex 1000 nM | 72 | N/A | 79 | N/A |
| Dex 1000 nM + BIRB 100 nM | 84* | 1.0 | 84 | 1.0 |
| Dex 1000 nM + BIRB 1000 nM | 84* | 1.0 | 85 | 1.0 |

Data presented as means. *, **, *** = significantly above dex alone (2-way ANOVA where p<0.05, p<0.01 and p<0.001 respectively).

**Supplementary table 4.** Percent inhibition of IL-1β induced IL-6 production from NHLFs and primary fibroblasts by dexamethasone and BIRB-796 alone or in combination.

| **Condition** | **NHLF** | | **Primary** | |
| --- | --- | --- | --- | --- |
|  | **% Inhibition** | **Interaction Ratio** | **% Inhibition** | **Interaction Ratio** |
| Dex 0.1nM | 41 | N/A | 10 | N/A |
| Dex 0.1 nM + BIRB 100 nM | 40 | 0.5 | 38 | 0.9 |
| Dex 0.1 nM + BIRB 1000 nM | 63 | 0.8 | 63 | 1.1 |
| Dex 1nM | 85 | N/A | 57 | N/A |
| Dex 1 nM + BIRB 100 nM | 72 | 0.8 | 64 | 0.9 |
| Dex 1 nM + BIRB 1000 nM | 76 | 0.8 | 75*** | 0.9 |
| Dex 10 nM | 89 | N/A | 87 | N/A |
| Dex 10 nM + BIRB 100 nM | 89 | 0.9 | 90 | 1.0 |
| Dex 10 nM + BIRB 1000 nM | 88 | 0.9 | 93 | 1.0 |
| Dex 100 nM | 90 | N/A | 92 | N/A |
| Dex 100 nM + BIRB 100 nM | 92 | 1.0 | 94 | 1.0 |
| Dex 100 nM + BIRB 1000 nM | 91 | 0.9 | 95 | 1.0 |
| Dex 1000 nM | 91 | N/A | 92 | N/A |
| Dex 1000 nM + BIRB 100 nM | 92 | 1.0 | 96 | 1.0 |
| Dex 1000 nM + BIRB 1000 nM | 91 | 0.9 | 96 | 1.0 |

Data presented as means. *** = significantly above dex alone (2-way ANOVA where p<0.001 respectively).
